# Role Played by the Passage of Time in Reversal Learning

**DOI:** 10.3389/fnbeh.2018.00075

**Published:** 2018-04-24

**Authors:** Estelle H. F. Goarin, Nura W. Lingawi, Vincent Laurent

**Affiliations:** Decision Neuroscience Laboratory, School of Psychology, University of New South Wales Sydney, Sydney, NSW, Australia

**Keywords:** reversal learning, extinction learning, inhibition, spontaneous recovery, pavlovian conditioning, instrumental conditioning, discrimination training

## Abstract

Reversal learning is thought to involve an extinction-like process that inhibits the expression of the initial learning. However, behavioral evidence for this inhibition remains difficult to interpret as various procedures have been employed to study reversal learning. Here, we used a discrimination task in rats to examine whether the inhibition produced by reversal learning is as sensitive to the passage of time as the inhibition produced by extinction. Experiment 1 showed that when tested immediately after reversal training, rats were able to use the reversed contingencies to solve the discrimination task in an outcome-specific manner. This ability to use outcome-specific information was lost when a delay was inserted between reversal training and test. However, interpretation of these data was made difficult by a potential floor effect. This concern was addressed in Experiment 2 in which it was confirmed that the passage of time impaired the ability of the rats to use the reversed contingencies in an outcome-specific manner to solve the task. Further, it revealed that the delay between initial learning and test was not responsible for this impairment. Additional work demonstrated that solving the discrimination task was unaffected by Pavlovian extinction but that the discriminative stimuli were able to block conditioning to a novel stimulus, suggesting that Pavlovian processes were likely to contribute to solving the discrimination. We therefore concluded that the expression of reversal and extinction learning do share the same sensitivity to the effect of time. However, this sensitivity was most obvious when we assessed outcome-specific information following reversal learning. This suggests that the processes involved in reversal learning are somehow distinct from those underlying extinction learning, as the latter has usually been found to leave outcome-specific information relatively intact. Thus, the present study reveals that a better understanding of the mechanisms supporting reversal training requires assessing the impact that this training exerts on the content of learning rather than performance *per se*.

## Introduction

Pavlovian extinction is commonly used to study how animals detect and adapt to changes in their environment (Delamater, 2004). In a typical extinction task, an initially neutral stimulus is trained to reliably predict the occurrence of a motivationally significant outcome. The stimulus-outcome contingency established by such training is later revealed by the capacity of the stimulus to elicit on its own various behavioral responses that reflect the properties of the predicted outcome. Extinction breaks the contingency by repeatedly presenting the stimulus in the absence of any consequence. As a result, the stimulus loses its capacity to trigger any responding. Reversal learning is another task that allows determining how animals adjust to change in stimulus-outcome contingencies (Delamater, 2007b). In this task, two stimuli are trained to reliably predict two distinct outcomes. Then, the two stimulus-outcome contingencies are reversed such that each stimulus now predicts the other outcome. Although much progress has been made in describing the psychological and neural mechanisms underlying extinction (Duvarci and Pare, 2014), little is known about those mediating reversal learning.

It is generally accepted that reversal training involves learning the reversed stimulus-outcome contingencies as well as inhibiting the original ones (Rescorla, 2007). The latter process of inhibition is essentially identical to that driving extinction learning and thereby, a wealth of evidence indicates that reversal and extinction learning share some key characteristics. Both require activity in similar brain regions such as the amygdala, the medial prefrontal cortex and the orbitofrontal cortex (Schiller and Delgado, 2010). Further, both appear to leave intact the originally learned stimulus-outcome contingencies. For instance, re-acquisition of the original contingencies following extinction or reversal learning occurs faster than their initial acquisition (Ricker and Bouton, 1996; Kangas and Bergman, 2014). Extinction or reversal of the original contingencies do not prevent these contingencies from guiding choice between actions in an outcome-specific manner (Rescorla, 1992a; Delamater, 1996). Finally, the responding elicited by the original contingencies spontaneously recover with the passage of time (Rescorla, 2007; Scarlet et al., 2009). Although convincing, the interpretation of these findings is somewhat complicated by the variety of procedures employed to investigate reversal learning. For example, many studies (Rescorla, 2007; Burke et al., 2009; Schiller and Delgado, 2010) used a procedure during which a motivational outcome is initially predicted by one stimulus (S1) but not another (S2). The arrangement is then reversed such that the outcome is now predicted by S2 and not S1. A first issue with such procedure is that it provides explicit extinction to S1 in the reversal stage as the stimulus is presented without any consequence. A second issue is that S2 may undergo latent inhibition during the first stage. Recently, latent inhibition and extinction have been shown to involve a similar form of inhibitory learning that is encoded and retrieved in the medial prefrontal cortex (Lingawi et al., 2017). Thus, some of the behavioral and neural commonalities previously described may not necessarily reflect those underlying extinction and reversal learning *per se*.

The present series of experiments therefore aimed at further investigating whether reversal learning shares similar behavioral characteristics with extinction learning. The critical feature of these experiments is in using a design that excluded a potential role for latent inhibition and explicit extinction. Thus, rats initially learned that two stimuli predicted the delivery of two distinct food outcomes. Specifically, each stimulus signaled that one of two available instrumental responses delivered a particular outcome. The contingencies established by this training were then reversed. Choice tests were administered immediately after reversal training to determine which contingency controlled instrumental behavior. Additional choice tests were given several days after reversal training to determine whether the learning produced by this training is sensitive to the passage of time in a similar manner as extinction learning. Importantly, all choice tests were conducted following an outcome-devaluation procedure in order to assess the relative impact on the outcome-specific contingencies established during initial and reversal training.

## Materials and Methods

### Subjects

The subjects were 48 experimentally naive female and male Long-Evans rats (at least 12-weeks old) bred in the Decision Neuroscience Laboratory at the University of New South Wales (Sydney, NSW, Australia). Half of the animals in Experiment 1 were female and the other half were male. Half of the animals in each group of Experiment 2 were female and the other half were male. All animals were housed in plastic boxes (two to four rats per box) located in a climate-controlled colony room and were maintained on a 12 h light/dark cycle (lights on between 7 am and 7 pm). Three days before the behavioral procedures, the rats were handled daily and were put on a food deprivation schedule. This schedule involved weighing the rats on a daily basis and providing an amount of food each day that maintained them at around 85% of their *ad libitum* feeding weight. The food was made available at least 1 h after each behavioral session. The Animal Ethics Committee at the University of New South Wales approved all experimental procedures. All these procedures were conducted between 7 am and 7 pm.

### Behavioral Apparatus

Training and testing took place in 16 Med Associates (St. Albans, VT, USA) operant chambers enclosed in sound- and light-resistant shells. A fan was attached to each shell and provided a background noise of approximately 60 dB. Each operant chamber was equipped with a pump fitted with a syringe that could deliver 0.1 ml of a 20% sucrose solution into a recessed magazine. A pellet dispenser individually delivered grain food pellets (45 mg; BioServe Biotechnologies). The chambers contained two retractable levers that could be inserted to the left and right side of the magazine. An infrared photobeam crossed the magazine opening, allowing for the detection of head entries. The chambers were also equipped with a white noise generator (90 dB), a Sonalert that delivered a 3 kHz pure tone (90 dB) and a 28 V DC mechanical relay that was used to deliver a 2 Hz clicker stimulus (90 dB). The white noise, the pure tone and the clicker served as the three auditory stimuli used in the experiments. One visual stimulus consisted of a house-light (3 W, 24 V) located on the end wall opposite the magazine. A second visual stimulus involved two lights mounted on either side of the magazine that were flashing at the same time and frequency (2 Hz). A set of two microcomputers running proprietary software (Med-PC; MED Associates) controlled all experimental events and recorded magazine entries and lever presses. Outcome devaluation was conducted in a separate room that contained 16 distinct plastic boxes (width: 26 cm; depth: 47.6 cm; height: 20.3 cm).

### Behavioral Procedures

#### Experiment 1

##### Overview

**Figure 1:**
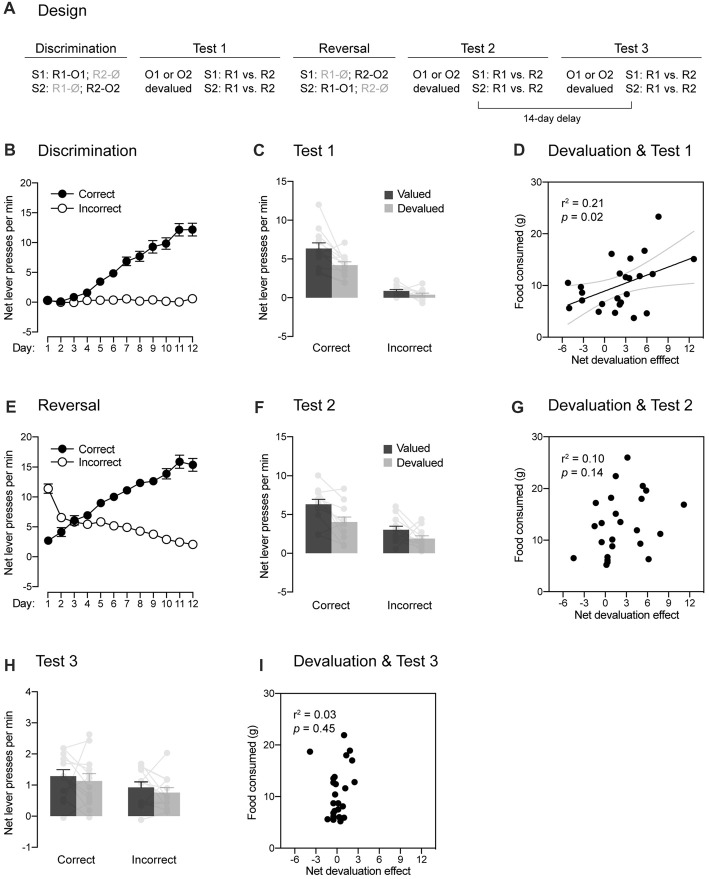
Within-subject demonstration of the role played by the passage of time in reversal learning. **(A)** Design of the experiment. Abbreviations—S1/S2: noise or clicker stimuli (counterbalanced); R1/R2: left or right lever press responses (counterbalanced); O1/O2: food pellet or sucrose solution outcomes (counterbalanced); Ø: no outcome. **(B)** Discrimination training occurred without incident, as rats learned that stimuli signaled which response was reinforced (Correct) and which one was not (Incorrect). **(C)** The specific contingencies established during discrimination training were successfully retrieved. Performance was higher on the correct response than on the incorrect one. Further, a stimulus signaling a valued outcome elicited more correct responding than a stimulus signaling a devalued outcome. Individual performance is indicated in light gray full circles. **(D)** The more an outcome had been consumed during the sensory-specific satiety stage, the less the correct response was performed. **(E)** Reversal training went smoothly and rats learned that the stimuli signaled which response was reinforced (Correct) and which one was not (Incorrect). **(F)** The reversed contingencies were successfully retrieved. Performance was higher on the correct response than on the incorrect one. However, the initial contingencies slightly interfered with the expression of the reversed contingencies: outcome-devaluation generally reduced instrumental responding. Individual performance is indicated in light gray full circles. **(G)** Consistent with this interference, there was no relationships between how much an outcome had been consumed across the sensory-specific satiety stage and responding on the correct response. **(H)** The passage of time abolished the ability of the reversed contingencies to control behavior. Although performance on the correct response remained marginally higher than that on the incorrect one, outcome-devaluation failed to influence instrumental responding. Individual performance is indicated in light gray full circles. **(I)** There was no relationship between the amount of food consumed across the sensory-specific satiety stage and responding on the correct response. Error bars denote ±1 SEM.

**Figure 2:**
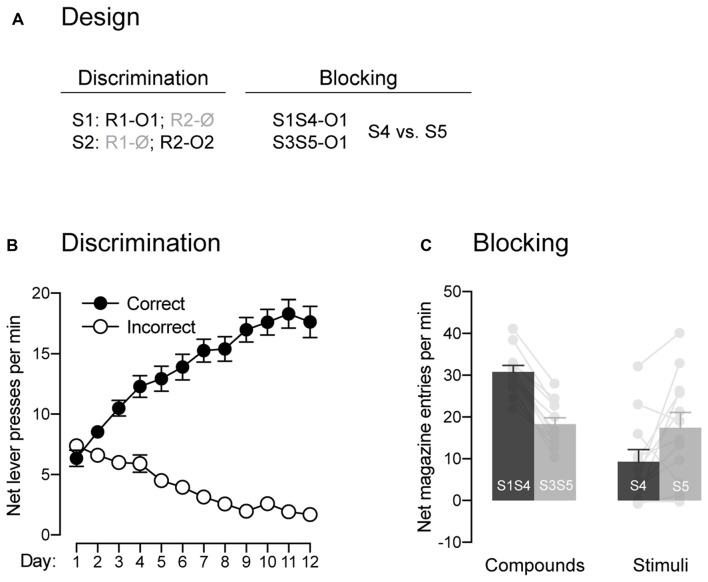
The signaling stimuli produce blocking. **(A)** Design used to assess the blocking phenomenon. Abbreviations—S3/S4/S5: tone, house-light and flashing lights (visual stimuli counterbalanced; other abbreviations are as described before. **(B)** Discrimination retraining was successful, as rats learned which stimulus signaled which response was reinforced (Correct) and which one was not (Incorrect). **(C)** The pre-training stimulus S1 blocked conditioning to the novel stimulus S4. Individual performance is indicated in light gray full circles. Error bars denote ±1 SEM.

Experiment 1 used a within-subject design (Figure 1A) to examine whether responding to reversed contingencies is influenced by the passage of time. A set of naïve rats received magazine, instrumental training followed by discrimination training. The latter involved learning that two distinct stimuli predicted which of two distinct instrumental responses earned one of two distinct food outcomes. Successful discrimination training was then assessed during a test administered immediately after a sensory-specific satiety manipulation. Then, all rats received reversal training where the previously learned contingencies were reversed. Successful reversal learning was assessed 1 day later during a test immediately administered after a sensory-specific satiety manipulation. A similar test was conducted 14 days later to examine the effect of the passage of time on reversal learning. Finally, all rats were submitted to a blocking procedure (Figure 2A) that aimed to determine some of the associative relationships controlling the behavior previously observed.

##### Magazine Training

All rats were administered a single session of magazine training during which they were placed in the operant chambers and received 20 deliveries of grain pellets and 20 deliveries of sucrose on a 60 s variable time (VT) schedule. During this training, both levers were retracted.

##### Instrumental Training

Following magazine training, the rats received two consecutive days of instrumental training during which the left and right lever press responses (R1 and R2) were trained to deliver the two food outcomes (O1 and O2; grain pellets and sucrose) in separate daily sessions (R1 → O1 and R2 → O2). The order of the sessions was counterbalanced, as were the response-outcome relationships. Lever pressing was continuously reinforced; i.e., each response earned one outcome. Each session ended when 40 outcomes were earned or when 60 min had elapsed. There was a 30-min delay between each session, during which the animals were placed back in their home-boxes.

##### Discrimination Training

The day after the end of instrumental training, rats received single lever discrimination training across two consecutive days. There were two separate sessions per day. In each session, one of the previously trained levers (R1 or R2) was made available and two auditory stimuli (S1 and S2; clicker and noise) were presented 12 times each for 60 s. Overall, in the presence of S1, pressing R1 earned O1 and pressing R2 earned nothing (S1: R1 → O1, R2 → Ø). Conversely, in the presence of S2, R2 earned O2 and pressing R1 earned nothing (S2: R1 → Ø, R2 → O2). The responses were reinforced on a VI 30 s schedule. The order of the sessions was fully counterbalanced as were the identities of the stimuli, responses and outcomes involved in each sequence. The order of the stimuli was varied between four sets of pseudorandom orders. The inter-trial interval (ITI) between each stimulus presentation was set at 15-s on the first day and was extended to 90-s on the second day. Once single lever discrimination training was completed, rats received a single daily discrimination training session across the next 10 days. In each session, the two levers (R1 and R2) were available and the two previously used auditory stimuli were presented 12 times each for 60 s. The identity of the response that was reinforced during a particular stimulus was identical to that used during the single lever discrimination training. That is, in the presence of S1, pressing R1 earned O1 and pressing R2 earned nothing (S1: R1 → O1, R2 → Ø). Conversely, in the presence of S2, R2 earned O2 and pressing R1 earned nothing (S2: R1 → Ø, R2 → O2). Reinforcement remained on a VI 30-s schedule and the ITI was kept at 90 s. The order of the stimuli varied between four sets of pseudorandom orders.

##### Devaluation and Test

Across the last 2 days of discrimination training, all animals were habituated to the devaluation cages. This was done by placing the animals in the cages for 1 h (one animal per cage). Twenty-four hours after the final day of discrimination training, the animals were returned to the devaluation cages and were given 1 h access to one of the two outcomes (O1 or O2). The identity of the devalued outcome was fully counterbalanced (i.e., half of the rats were devalued with the grain pellets and the other half with the sucrose solution). Immediately after devaluation, the rats were placed into the operant chambers for test. During this test, the two responses, R1 and R2, were available and the two auditory stimuli, S1 and S2, were presented six times each for 60-s with an ITI of 90-s. No outcomes were delivered during this test. The following day rats again received devaluation followed by another test. The procedure was identical to that just described except that the other outcome was devalued (i.e., the rats that had been devalued with the grain pellets were now devalued with the sucrose solution, and conversely).

##### Reversal Discrimination Training

Following the second test, rats were submitted to three consecutive days of discrimination training in the manner described previously. The aim was to reinstate responding to pre-test levels. Then, all rats received reversal discrimination training across the next 12 days. This training was identical to the initial discrimination training except that the single lever training sessions were omitted and the contingencies were reversed. Thus, in the presence of S1, pressing R2 earned O2, and pressing R1 earned nothing (S1: R1 → Ø, R2 → O2). Conversely, in the presence of S2, pressing R1 delivered O1, and pressing R2 earned nothing (S2: R1 → O1 R2 → Ø).

##### Devaluation and Test

Following reversal discrimination training, all rats received two consecutive days of devaluation and test in the manner described before. Fourteen days later, another round of devaluation and test was given.

##### Blocking

Following the last test, rats were retrained across 12 days on the contingency used during the initial discrimination training; S1: R1 → O1, R2 → Ø; S2: R1 → Ø, R2 → O2. On the final day of this retraining, rats were also pre-exposed to three new stimuli: a tone (S3), the house-light and the flashing light (S4 and S5). The day after retraining, rats were given a single blocking session. During that session, one of the previously trained auditory stimuli (S1) was presented in compound with one of the novel visual stimuli (S4). Another compound stimulus consisted in presenting the novel auditory stimulus (S3) with the other novel visual stimulus (S5). The identity of S4 and S5 was fully counterbalanced. Each compound ended with the delivery of the outcome (O1) that was predicted by the already trained auditory stimulus S1. Each compound was presented in a pseudorandom order seven times for 30 s. At the end of these presentations, the two visual stimuli (S4 and S5) were experienced once on their own. Throughout the session, an ITI ranging between 1 min and 5 min and averaging 3 min was used.

### Experiment 2

#### Overview

**Figure 3:**
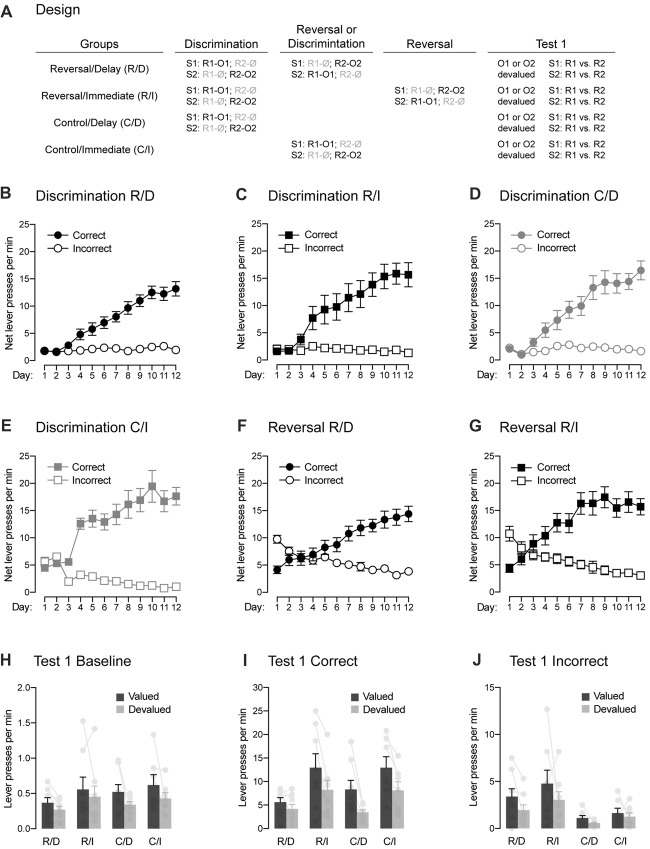
Between-subject demonstration of the role played by the passage of time in reversal learning. **(A)** Design of the experiment. Abbreviations are as described before. **(B–E)** Discrimination training occurred smoothly in all groups. All rats learned that the stimuli signaled which response was reinforced (Correct) and which one was not (Incorrect). **(F,G)** The two groups submitted to reversal training successfully learned the change in the original contingencies. **(H)** Outcome-devaluation reduced baseline instrumental performance in the absence of the stimuli. **(I)** In the two control groups, a stimulus signaling a valued outcome elicited more correct responding than a stimulus signaling a devalued outcome. The same behavior was observed in rats that had received reversal training and that were tested immediately after that training. The effect of outcome devaluation was significantly reduced in rats that had received reversal training and that were tested 2 weeks later, suggesting that the passage of time increased the interference of the initial contingencies over the reversed contingencies. Individual performance is indicated in light gray full circles. **(J)** All groups displayed higher performance on the incorrect response that was associated with a valued outcome compared to that associated with a devalued one. Rats that had reversal training had higher levels of performance on the incorrect response that control rats, suggesting that they retained information about the initial contingencies. Individual performance is indicated in light gray full circles. Error bars denote ±1 SEM.

**Figure 4:**
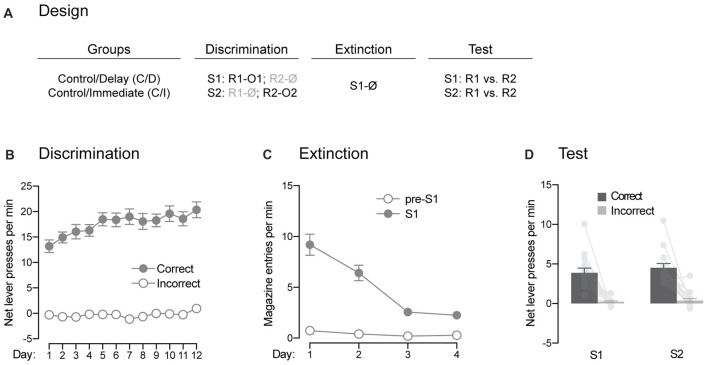
Pavlovian extinction spares the ability of the signaling stimuli to guide instrumental responding. **(A)** Design of the extinction procedure administered to the control rats. Abbreviations are as described before. **(B)** Discrimination retraining was successful, as rats learned which stimulus signaled which response was reinforced (Correct) and which one was not (Incorrect). **(C)** Extinction training was also successful as the levels of magazine entries elicited by the stimulus gradually decreased across days. **(D)** Extinction had no effect on the ability of the stimuli to signal which response was reinforced and which one was not. Individual performance is indicated in light gray full circles. Error bars denote ±1 SEM.

**Figure 5:**
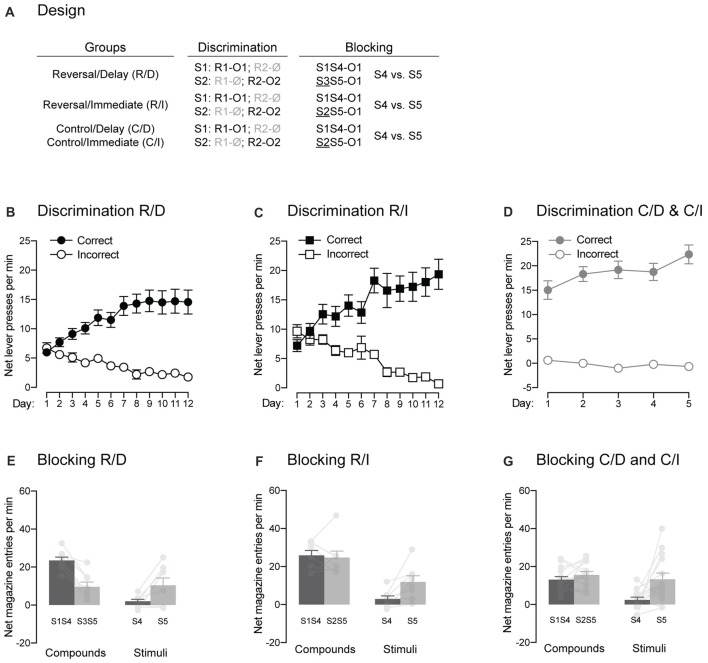
The signaling stimuli produce two forms of blocking. **(A)** Design of the two forms of blocking employed. Abbreviations are as described before. **(B–D)** Discrimination retraining was successful, as all rats learned which stimulus signaled which response was reinforced (Correct) and which one was not (Incorrect). **(E–G)** The pre-training stimulus S1 blocked conditioning to the novel stimulus S4 regardless of the blocking design employed. Individual performance is indicated in light gray full circles. Error bars denote ±1 SEM.

Experiment 2 used a between-subject design (Figure 3A) to examine whether responding to reversed contingencies is influenced by the passage of time. Four groups of rats received magazine, instrumental training and discrimination training. Then, two of these groups (Groups Reversal/Delay and Reversal/Immediate) underwent reversal training of the original discrimination. One group was tested immediately after this training (Group Reversal/Immediate) whereas the other was tested 12 days later (Group Reversal/Delay). The remaining two groups (Groups Control/Delay and Control/Immediate) were controls, they did not receive reversal training but were tested either 12 (Group Control/Immediate) or 24 days (Group Control/Delay) after their original discrimination training. After test, we assessed whether extinction of Pavlovian stimuli would prevent the expression of the learning produced by discrimination training (Figure 4A). Finally, all rats were submitted to two distinct blocking procedures (Figure 5A) that aimed to determine some of the associative relationships controlling the behavior previously observed.

#### Magazine Training and Instrumental Training

All rats received 1 day of magazine training followed by 2 days of instrumental training in the manner previously described.

#### Discrimination Training

The day after instrumental training, three groups of rats (Groups Reversal/Delay, Reversal/Immediate and Control/Delay) were submitted to discrimination training. As before, this training started with two consecutive days of single lever discrimination training followed by 10 days of single daily discrimination training sessions. The parameters were identical to those previously described. Thus, rats learned that in the presence of S1, pressing R1 earned O1, and pressing R2 earned nothing (S1: R1 → O1, R2 → Ø). Conversely, in the presence of S2, R2 earned O2, and pressing R1 earned nothing (S2: R1 → Ø, R2 → O2). Once that initial training was completed, rats in Group Control/Delay did not receive any further training before their first test. Rats in Group Control/Immediate started the discrimination training just described once the other groups had finished that training (i.e., 12 days later).

#### Reversal Discrimination Training

The day after completing the initial discrimination training, rats in Group Reversal/Delay started reversal discrimination training. This training was as described previously and consisted of reversing the previous contingencies. Thus, in the presence of S1, pressing R2 now delivered O2, whereas pressing R1 earned nothing (S1: R1 → Ø, R2 → O2). Conversely, in the presence of S2, pressing R1 now delivered O1, and pressing R2 delivered nothing (S2: R1 → O1; R2 → Ø). Rats in Group Reversal/Immediate also received reversal discrimination training but did so the day after rats in Group Reversal/Delay finished theirs. That is, they underwent reversal discrimination training 12 days after the initial discrimination training.

#### Devaluation and Test

Following the last day of reversal discrimination training administered to rats in Group Reversal/Immediate, rats in all groups received two consecutive days of devaluation and test in the manner described before. Once again, the animals had been habituated to the devaluation cages prior to receiving their first devaluation stage. The day after the second test, all rats were retrained on the initial discrimination contingency for 12 days.

#### Pavlovian Extinction and Test

The day after initial discrimination retraining, rats in Groups Control/Delay and Control/Immediate received daily sessions of Pavlovian extinction across four consecutive days. The levers were retracted during these sessions. Each session consisted of presenting one of the pre-trained stimuli (S1) alone eight times. The stimulus lasted 30-s. An ITI ranging between 1 min and 5 min and averaging 3 min was used. Following the last day of extinction, all rats received a single test during which the two trained responses (R1 and R2) were available. Both S1 and S2 were presented eight times each. Each presentation lasted 30-s and a fixed ITI of 90-s was used. Following this, all rats were again submitted to discrimination retraining for five consecutive days.

#### Blocking

The day after their first discrimination retraining, rats in Groups Reversal/Delay and Reversal/Immediate received a single blocking session. This session was identical to that described in Experiment 1 for half of the rats. Thus, two compounds, S1S4 and S3S5, ended with the delivery of outcome O1 and the session ended with a single presentation of S4 and S5 on their own. The other of half rats received a modified blocking procedure. In that procedure, the two compounds included one of the previously trained auditory stimuli (S1 and S2) with one of the new visual stimuli (S4 and S5). The two compounds, S1S4 and S2S5, ended with the delivery of outcome O1. At the end of the session, the visual stimuli, S4 and S5, were presented on their own. This modified blocking procedure was also administered to rats in Groups Control/Delay and Control/Immediate, once they had finished their second round of discrimination retraining. All rats had been pre-exposed to the new stimuli (S3, S4 and S5 or S4 and S5) used during the blocking stage.

### Statistical Analysis

The number of lever presses and entries into the magazine where the food was delivered were recorded by the Med Associate software. During the discrimination, reversal and test stages, baseline responding was defined as the mean number of lever presses per minute on both responses when stimuli were absent. Unless indicated otherwise, this responding was subtracted to reveal the net increase in performance; i.e., the net effect of the stimuli. Overall, baseline responding remained very low throughout the various stages and appropriate analyses were conducted to ensure it was not affecting net performance. A similar approach was adopted during the blocking stages where baseline responding was defined as the mean number of magazine entries per minute when the stimuli were absent. The differences between groups or stimuli were analyzed by means of planned contrasts with the Bonferroni inequality used to control the experiment-wise error rate. Within-session changes of responding were assessed by a planned linear trend analysis. All these procedures and analyses have been described by Hays (1963) and were conducted in the PSY software (School of Psychology, The University of New South Wales, Australia). The Type I error rate was controlled at 0.05 for each contrast tested. Measures of effect size [partial eta-squared *η*^2^] are also reported for each statistically significant comparison (*η*^2^ = 0.01 is a small effect, *η*^2^ = 0.06 a medium effect and *η*^2^ = 0.14 a large effect. All correlative analyses were conducted using the Pearson product moment correlation.

## Results

### Experiment 1

Experiment 1 used a within-subject design (Figure 1A) to examine whether responding to reversed contingencies is influenced by the passage of time. A set of naive rats initially received discrimination training during which two stimuli (S1 and S2) signaled the delivery of two distinct response-contingent food outcomes (O1 and O2). Specifically, S1 signaled that one lever press response (R1) delivered O1, whereas S2 signaled that another lever press response (R2) earned O2 (i.e., S1: R1 → O1, R2 → Ø; S2: R1 → Ø, R2 → O2). The encoding of these contingencies was then tested using a sensory-specific satiety procedure during which the value of O1 (or O2) was reduced by giving free access to that outcome for 1 h. Immediately after the procedure, choice between the two trained responses (R1 and R2) was assessed in the presence of either S1 or S2. No outcomes were delivered during that choice test to prevent any further learning. If the discrimination training had established specific contingencies, we would expect choice to be altered by devaluation such that a stimulus trained to signal a particular outcome would be less likely to increase instrumental performance if that outcome had been devalued. Following that first round of testing, rats were retrained on the initial discrimination before receiving reversal training. This training was similar to the initial one except that the contingencies previously established were reversed. Thus, S1 now signaled that R2 delivered O2, whereas S2 signaled that R1 delivered O1 (i.e., S1: R1 → Ø, R2 → O2; S2: R1 → O1, R2 → Ø). Successful learning of the reversed contingencies was then evaluated by the sensory-specific satiety and choice test procedure described before. Finally, all rats were tested again 14 days later to determine whether the passage of time influences responding after reversal training.

#### Discrimination Training

Four rats (one female and three males) were excluded for failing to learn the initial discrimination, leaving 12 animals in the final statistical analyses. The data from the initial discrimination training are presented in Figure 1B. They are plotted as the mean net number of lever presses per minute when the response performed was reinforced (“Correct”) or not (“Incorrect”) in the presence of the two stimuli. Thus, R1 was reinforced and labeled as the correct response in the presence of S1 whereas it was not reinforced and was labeled as the incorrect response in the presence of S2. Conversely, R2 was labeled as the correct response in the presence of S2 and as the incorrect response in the presence of S1. The statistical analyses revealed that discrimination training was successful as responding increased across days (*F*_(1,11)_ = 122.65, *p* < 0.05, *η*^2^ = 0.92), it was higher on the correct response than on the incorrect one (*F*_(1,11)_ = 102.87, *p* < 0.05, *η*^2^ = 0.9) and the difference between the two responses grew larger as training progressed (*F*_(1,11)_ = 86.33, *p* < 0.05, *η*^2^ = 0.89).

#### Choice Test 1

The learning produced by discrimination training was then assessed through a choice test conducted after a sensory-specific satiety manipulation. Performance during this test is shown in Figure 1C as the mean net number of lever presses per minute on the correct and incorrect responses in the presence of the stimuli. Further, responding on the correct and incorrect responses was separated according to whether the responses used to deliver an outcome that was now devalued (Devalued) or not (Valued). Overall, the statistical analysis at test revealed more responding on the correct response than the incorrect one (*F*_(1,11)_ = 97.98, *p* < 0.05, *η*^2^ = 0.9) and higher performance on the valued response than the devalued one (*F*_(1,11)_ = 12.76, *p* < 0.05, *η*^2^ = 0.54). Importantly, responding on the correct and incorrect response depended on whether or not the outcome that they used to earn had been devalued or not (*F*_(1,11)_ = 5.26, *p* < 0.05, *η*^2^ = 0.32). Thus, devaluation reduced responding on the correct response (*F*_(1,11)_ = 10.02, *p* < 0.05, *η*^2^ = 0.48) but it failed to do so on the incorrect response (*F* < 2.98). It should be noted that the size of the devaluation effect on the correct response was perhaps smaller than expected. However, this should not be taken as indication that outcome-specific encoding was weak. For instance, we found that the more the animals had consumed the outcome during the sensory-specific satiety stage, the less they performed the correct response (Figure 1D; *r* = 0.46, *p* < 0.05). Thus, the efficacy of the sensory-specific manipulation, rather than the strength outcome-specific encoding, may be responsible for the size of the devaluation effects observed here. Regardless, the results indicate that animals successfully used the stimuli to select the response that was reinforced. Further, the contingencies established during discrimination training were encoded in an outcome-specific manner as their expression depended on outcome value.

#### Reversal Training

Rats were briefly retrained on the initial discrimination before being submitted to reversal training during which the initial contingencies were reversed. Accordingly, the data presented in Figure 1E labeled R2 as the correct response in the presence of S1 and conversely, R1 was the correct response in the presence of S2. Inspection of the figure reveals that the initial tendency of animals to respond according to the initial contingencies was quickly reversed such that performance became consistent with the new and reversed contingencies. This was confirmed by the statistical analysis that revealed higher performance on the correct response than the incorrect one (*F*_(1,11)_ = 106.85, *p* < 0.05, *η*^2^ = 0.91), an increase of responding across days (*F*_(1,11)_ = 26.69, *p* < 0.05, *η*^2^ = 0.71) and a significant interaction between these two factors (*F*_(1,11)_ = 208.19, *p* < 0.05, *η*^2^ = 0.95).

#### Choice Test 2

The learning produced by reversal training was then assessed by administering a choice test after a sensory-specific satiety manipulation. The data are presented in Figure 1F in the manner previously described. The statistical analysis revealed higher responding on the correct response than the incorrect response (*F*_(1,11)_ = 20.66, *p* < 0.05, *η*^2^ = 0.65) as well as higher performance on the valued response than the devalued one (*F*_(1,11)_ = 22.07, *p* < 0.05, *η*^2^ = 0.67). However, unlike what was observed after the initial discrimination training, the change in outcome value failed to differentially affect performance on the correct and incorrect response (*F* < 1.29). This suggests that both the initial and reversed contingencies were competing to influence behavior. Consistent with this, we failed to obtain a positive correlation (Figure 1G) between the amount of food consumed during the sensory-specific manipulation and the devaluation effect produced on correct responding (*p* = 0.14). Nevertheless, the present results indicate that the learning produced by reversal training dominated the one produced by initial discrimination training at the behavioral level: performance was higher on the correct response than the incorrect one and responding on the devalued response was lower than that of the valued response. Further, it is worth noting that separate analyses revealed an effect of devaluation on correct responding (*F*_(1,11)_ = 13.74, *p* < 0.05, *η*^2^ = 0.56) but not incorrect responding (*F* < 1.7). Thus, animals successfully learned the reversal of the initial contingencies and the reversed contingencies were encoded in outcome-specific manner.

#### Choice Test 3

To determine whether the passage of time interferes with responding after reversal training, rats received a last round of sensory-specific satiety and choice test 14 days after the last one. The results are shown in Figure 1H in the manner previously described, where “correct” and “incorrect” refer to the reversed contingencies. The statistical analysis revealed higher responding for the correct response than the incorrect one (*F*_(1,11)_ = 12.61, *p* < 0.05, *η*^2^ = 0.53), suggesting that the learning produced by reversal training was dominating that produced by initial training. However, the analysis failed to reveal any effect of devaluation (*F* < 1) and responding on the correct and incorrect responses was not influenced by devaluation (*F* < 0.1). Accordingly, we also failed to obtain a positive correlation (Figure 1I) between the amount of food consumed during the sensory-specific manipulation and the devaluation effect produced on correct responding (*p* = 0.44). The passage of time had therefore a massive impact on responding such that the animals were no longer able to use the outcome-specific information to direct their behavior. This inability is consistent with the view that animals learned to inhibit the initial contingencies during reversal training and that this inhibitory learning wanes with the passage of time, increasing interference of the initial contingency over the reversed one at the behavioral level. However, it is important to note that performance during the test were low and it remains possible that a floor effect may have masked an effect of the outcome devaluation on instrumental responding.

#### Blocking

In the present behavioral task, the animals are required to perform one of two available responses in order to gain access to the outcomes signaled by the two trained stimuli. Previous studies have demonstrated that such behavior can be controlled by various types of associations that are not mutually exclusive (Colwill and Rescorla, 1988, 1990; Bradfield and Balleine, 2013). Critical to our interest was whether or not the behavior presently produced does involve Pavlovian associations, those established between a stimulus and the outcome that it signals. One key characteristic of a Pavlovian stimulus is its capacity to generate the phenomenon of blocking: it prevents the formation of an association between its outcome and a co-present neutral stimulus (Kamin, 1968). Thus, we used the blocking phenomenon to determine whether the present task produced Pavlovian associations between the two stimuli and the two outcomes (Figure 2A). Following the last round of test, animals were retrained on the initial contingencies, where S1 signaled that R1 delivered O1 and S2 signaled that R2 earned O2. The data across retraining are presented in Figure 2B and are plotted in the same manner as before. Retraining occurred smoothly as performance increased across days (*F*_(1,11)_ = 28.67, *p* < 0.05, *η*^2^ = 0.67), responding was higher on the correct response than the incorrect one (*F*_(1,11)_ = 231.36, *p* < 0.05, *η*^2^ = 0.95) and the difference between the two responses grew larger as training progressed (*F*_(1,11)_ = 261.64, *p* < 0.05, *η*^2^ = 0.96). Retraining was followed by a single blocking session during which two compounds signaled the delivery of outcome O1. One of these compounds was composed of the previously trained stimulus S1 and a novel stimulus S4. The other compound was composed of two novel stimuli, S3 and S5. If our task produced Pavlovian associations, we reasoned that the pre-trained S1 would block conditioning to the novel stimulus S4. This would be evidenced by lower levels of Pavlovian responses elicited by S4 relative to S5, as the latter stimulus was trained with a stimulus that had never been trained to signal O1. The results from the blocking stage are presented in Figure 2C and are plotted as the mean net number of entries in the magazine per minute across the various compounds and stimuli. The statistical analysis revealed that the compounds elicited more responding than the individual stimuli (*F*_(1,11)_ = 16.03, *p* < 0.05, *η*^2^ = 0.59) and that responding averaged across S1S4 and S4 alone did not differ from responding averaged across S3S5 and S5 alone (*F* < 1.24). Importantly, responding to S4 and S5 depended on whether they were presented in compound or not (*F*_(1,11)_ = 46.03, *p* < 0.05, *η*^2^ = 0.81). Thus, there was more responding to S4 when it was presented with the pre-trained S1 than to S5 when it was presented with the novel S3 (*F*_(1,11)_ = 173.02, *p* < 0.05, *η*^2^ = 0.94). However, S5 alone elicited more responding than S4 alone (*F*_(1,11)_ = 5.90, *p* < 0.05, *η*^2^ = 0.35), suggesting successful blocking to S4 by S1. The ability of S1 to produce blocking to S4 suggest that our task generated Pavlovian associations between the stimuli and the outcomes and that these associations were likely to contribute to the behavior observed.

### Experiment 2

The previous experiment revealed that rats were able to adjust their behavior to bring it in line with the reversed contingencies. This adjustment was evidenced by the choice test administered immediately after reversal training. This test showed that rats successfully used the signaling stimuli to select the response delivering food. It also showed that they had acquired knowledge about the specific outcomes signaled by the stimuli. For instance, a stimulus signaling a valued outcome triggered higher responding than a stimulus signaling a devalued outcome. Critically, the previous experiment also revealed that the behavioral adjustment produced by reversal training was sensitive to the passage of time. Evidence of this adjustment was lost with the insertion of a 2-week delay between reversal training and the choice test. This finding is consistent with the view that the initial contingencies were inhibited across reversal training and that this inhibition wanes with the passage of time. As a result, the re-emergence of the initial contingencies may have interfered with the expression of the reversed contingencies during the delayed test and thereby, disrupted choice during that test. There are, however, at least two major issues with the previous experiment. The first is that responding during the delayed test was very low, presumably due to extinction produced by the choice test conducted immediately after reversal training. This low level of responding raises the possibility that a floor effect may have masked evidence for adequate reversal behavior during the delayed test. The second issue is that our design confounded the role that a time interval may play when inserted between reversal training and a choice test and the one it may play when inserted between initial training and that same test. To address these issues, Experiment 2 used a between-subject design (Figure 3A) that included four distinct groups of rats. All groups underwent discrimination training during which S1 signaled that R1 delivered O1 whereas R2 earned nothing (S1: R1 → O1, R2 → Ø). Conversely, S2 signaled that R1 earned nothing whereas R2 delivered O2 (S2: R1 → Ø, R2 → O2). Then, two groups (Reversal/Delay and Reverse/Immediate) received reversal training: S1 now signaled that R2 delivered O2, whereas S2 signaled that R1 delivered O1 (i.e., S1: R1 → Ø, R2 → O2; S2: R1 → O1, R2 → Ø). Twenty-four hours after reversal training, one group (Reverse/Immediate) underwent a devaluation manipulation and a choice test in the manner previously described. By contrast, this devaluation and test were given 12 days later in the other group (Reversal/Delay) in order to test for the effect of the passage of time in reversal learning. The two remaining groups were control groups (Control/Delay and Control/Immediate) that did not receive reversal training. They were tested either 12 days or 24 days after the original discrimination training. Importantly, the delay between initial discrimination training and the choice test was identical in the two groups that had received reversal training (Reverse/Delay and Reverse/Immediate). Although they differed in terms of the time interval between the original discrimination training and reversal training, this difference was controlled by the addition of the two control groups (Control/Delay and Control/Immediate) in which performance was expected to be similar. Thus, any difference between the groups of rats that received reversal training would be due to the time interval inserted between this training and the choice test.

#### Discrimination Training

One animal (1 female) from group Control/Immediate was excluded for failing to learn the initial discrimination, yielding the following group numbers: Reverse/Delay, *n* = 8; Reverse/Immediate, *n* = 8; Control/Delay, *n* = 8; Control/Immediate, *n* = 7. The data for the initial discrimination training for each group are presented in Figures 3B–E and they are plotted in the manner previously described. The analyses of baseline responding in the absence of the stimuli revealed similar performance for rats in groups Reverse/Delay, Reverse/Immediate and Control/Delay (*F*s < 1.8). However, rats in group Control/Immediate exhibited higher baseline responding than those three groups (*F*_(1,27)_ = 27.71, *p* < 0.05, *η*^2^ = 0.49). The source of this difference remains unclear and reflected by abnormally high baseline performance of the Control/Immediate at the start of the training. Indeed, a separate analysis conducted across the last 7 days of the discrimination revealed no difference among the groups (*F*s < 0.4). As a result, performance across discrimination training was analyzed as before by comparing the mean net number of lever presses on the correct and incorrect responses. The statistical analyses revealed a significant increase of responding across days (*F*_(1,27)_ = 180.182, *p* < 0.05, *η*^2^ = 0.85), higher performance on the correct response than the incorrect one (*F*_(1,27)_ = 162.93, *p* < 0.05, *η*^2^ = 0.88) and the discrimination between these two responses grew larger as training progressed (*F*_(1,27)_ = 239.21, *p* < 0.05, *η*^2^ = 0.85). Importantly, this discrimination was not influenced by the group in which the animals were allocated (*F*s < 2.7) and there was no overall difference between groups (*F*s < 3.7). Thus, discrimination training was successful in all groups.

#### Reversal Training

Performance during reversal training are plotted in Figures 3F,G in the manner previously described. That is, the correct and incorrect responses were labeled according to the reversed contingencies: R2 was the correct response in the presence of S1 and conversely, R1 was the correct response in the presence of S2. Inspection of the figure revealed that the initial tendency of animals to respond according to the initial contingencies was quickly reversed such that performance became consistent with the new reversed contingencies. This was confirmed by the statistical analysis, as there was a significant increase in responding across days (*F*_(1,14)_ = 32.01, *p* < 0.05, *η*^2^ = 0.7) and performance on the correct response was higher than on the incorrect one (*F*_(1,14)_ = 40.81, *p* < 0.05, *η*^2^ = 0.71) and the discrimination between the two responses grew larger as training progressed (*F*_(1,14)_ = 199.202, *p* < 0.05, *η*^2^ = 0.93). Importantly, there was no difference between the two groups of rats (*F*s < 2.8), indicating similar and successful reversal learning of the initial contingencies.

#### Choice Test

The data from the choice test that assessed the effects of the signaling stimuli on instrumental responding are presented in Figures 3H–J. Baseline responding in the absence of the stimuli (Figure 3H) remained low and was similar among groups (*F*s < 1.9). However, performance on the valued response was found to be higher overall than that on the devalued response (*F*_(1,27)_ = 5.92, *p* < 0.05, *η*^2^ = 0.18) regardless of the group considered (*F*s < 0.6). As a result, we chose to analyze responding on the correct (Figure 3I) and incorrect (Figure 3J) responses in the presence of the stimuli without subtracting baseline responding. Overall, the analysis revealed similar responding whether reversal training had been administered or not (Reverse/Delay and Reversal/Immediate vs. Control/Delay and Control/Immediate; *F* < 0.8). It also revealed higher responding in the absence of a delay between training and test (Reverse/Immediate and Control/Immediate vs. Reverse/Delay and Control/Delay; *F*_(1,27)_ = 17.03, *p* < 0.05, *η*^2^ = 0.37). Importantly, performance on the correct response was generally higher than on the incorrect one (*F*_(1,27)_ = 150.71, *p* < 0.05, *η*^2^ = 0.73) and it was also higher on the valued response than the devalued one (*F*_(1,27)_ = 44.37, *p* < 0.05, *η*^2^ = 0.56). Critically, responding on the correct and incorrect response depended on outcome value and on whether or not animals had received reversal training (*F*_(1,27)_ = 12.24, *p* < 0.05, *η*^2^ = 0.14). Thus, we conducted additional and separate analyses for correct and incorrect responding.

The analysis conducted on incorrect responding (Figure 3J) revealed higher performance when the response was associated with a valued outcome than a devalued one (*F*_(1,27)_ = 13.03, *p* < 0.05, *η*^2^ = 0.27). Responding was marginally lower when a delay had been inserted between training and test (Reverse/Immediate and Control/Immediate vs. Reverse/Delay and Control/Delay; *F*_(1,27)_ = 4.67, *p* < 0.05, *η*^2^ = 0.11). Interestingly, rats that received reversal training displayed higher levels of responding than rats that had not received that training (*F*_(1,27)_ = 9.046, *p* < 0.05, *η*^2^ = 0.22) regardless of outcome value or the presence of a delay before test (*F*s < 1.9). This increase in responding is likely to reflect the influence of the initial contingencies on behavior. Regardless, the data of most interest are those obtained on correct responding (Figure 3I). Provided the results obtained in the previous experiment, we expected the devaluation manipulation to exert a stronger influence on correct responding in the two control groups and the reversed group with no delay than on the reversed group that had a delay inserted between reversal training and test. Accordingly, we found a similar effect of outcome devaluation in the two control groups (Control/Delay and Control/Immediate; *F* < 0.82) and between these two groups and the group Reverse/Immediate (*F* < 0.1). All three groups exhibited higher performance on the correct response that earned the valued outcome as opposed to one that delivered the devalued one (*F*_(1,20)_ = 59.57, *p* < 0.05, *η*^2^ = 0.74). Critically, the devaluation effect in these three groups was significantly larger than the one observed in group Reverse/Delay (*F*_(1,27)_ = 8.1, *p* < 0.05, *η*^2^ = 0.08), although this group still displayed lower responding on the devalued response than the value one (*F*_(1,7)_ = 12.89, *p* < 0.05, *η*^2^ = 0.65). The reduction in the effect of devaluation in group Reverse/Delay is consistent with the results obtained in the previous experiment and suggests that the passage of time allowed the initial contingencies to interfere and compete with the reverse contingencies to control behavior.

#### Pavlovian Extinction

The previous experiment suggested that direct associations between the trained stimuli and outcomes are established in our behavioral task. Here, we assessed the effects of extinction of these associations on the ability of the stimuli to guide instrumental responding (Figure 4A). Rats from the two control groups received additional discrimination training (Figure 4B). This retraining went smoothly as overall performance increased across days (*F*_(1,13)_ = 103.16, *p* < 0.05, *η*^2^ = 0.89), responding was higher on the correct response than the incorrect one (*F*_(1,13)_ = 175.14, *p* < 0.05, *η*^2^ = 0.93) and the discrimination between the two responses grew larger across days (*F*_(1,13)_ = 29.95, *p* < 0.05, *η*^2^ = 0.59). The rats then received extinction training during which the association between S1 and O1 was broken by repeatedly presenting S1 on its own (Figure 4C). Extinction was successful as the levels of magazine entries elicited by S1 gradually declined across training (*F*_(1,13)_ = 66.7, *p* < 0.05, *η*^2^ = 0.84). Although these levels were higher in the presence of the stimulus than in its absence (*F*_(1,13)_ = 87.3, *p* < 0.05, *η*^2^ = 0.87), the difference between the two periods got smaller as training progressed (*F*_(1,13)_ = 46.63, *p* < 0.05, *η*^2^ = 0.78). Following extinction training, we evaluated the ability of the extinguished (S1) and non-extinguished (S2) stimulus to drive instrumental responding according to the contingencies established during discrimination training (Figure 4D). The statistical analysis revealed higher performance on the correct response than the incorrect one (*F*_(1,13)_ = 59.47, *p* < 0.05, *η*^2^ = 0.82). There was no effect of extinction (*F* < 2.5) and both the extinguished S1 and the non-extinguished S2 were able to direct responding towards the correct response (*F* < 0.34). Thus, extinction of the stimulus-outcome contingencies established across discrimination training does not remove the ability of the stimuli to guide adequate instrumental responding in our task.

#### Blocking

Experiment 1 provided evidence for the establishment of direct associations between the trained stimuli and outcomes using the blocking phenomenon. Here, we sought to replicate this evidence (Figure 5A). Rats in group Reverse/Delay were first retrained on their initial discrimination contingencies. Retraining (Figure 5B) was successful as performance increased across days (*F*_(1,7)_ = 10.85, *p* < 0.05, *η*^2^ = 0.61), responding was higher on the correct response than the incorrect one (*F*_(1,7)_ = 39.72, *p* < 0.05, *η*^2^ = 0.85) and the discrimination between the two responses grew larger as training progressed (*F*_(1,7)_ = 26.83, *p* < 0.05, *η*^2^ = 0.79). The rats then underwent the same blocking procedure as used before: two compounds, S1S4 and S3S5, terminated in the delivery of O1 and the number of magazine entries elicited by S4 and S5 alone were later assessed. The results presented in Figure 5E fully replicated those found in Experiment 1. The compounds elicited more magazine entries than the stimuli (*F*_(1,7)_ = 42.06, *p* < 0.05, *η*^2^ = 0.86) and responding to S1S4 and S4 alone did not differ from that of S3S5 and S5 alone (*F* < 1.04). Importantly, responding to S4 and S5 depended on whether they were presented in compound or not (*F*_(1,7)_ = 76.09, *p* < 0.05, *η*^2^ = 0.92). Thus, there was more responding to S4 when it was presented with the pre-trained S1 than to S5 when it was presented with the novel S3 (*F*_(1,7)_ = 43.25, *p* < 0.05, *η*^2^ = 0.86). However, S5 alone elicited more responding than S4 alone (*F*_(1,11)_ = 5.76, *p* < 0.05, *η*^2^ = 0.45), suggesting successful blocking of S4 by S1.

One issue with the blocking results just described is that S1S4 elicited significantly more magazine entries than S3S5. Although this is not surprising given that S1 had been trained previously, it raised the possibility that any subsequent difference between S4 and S5 was due to a strong overshadowing effect of S1 upon S4, leading to lower responding to S4 compared to S5 (Holland, 1999). As a result, we employed a modified blocking design for rats in group Reverse/Immediate (Figure 5A). These rats were first retrained on their initial discrimination contingencies (Figure 5C). Retraining occurred smoothly even though overall performance failed to increase across days (*F* < 1.3), presumably because it was high early in training. Regardless, responding was higher on the correct response than the incorrect one (*F*_(1,7)_ = 35.19, *p* < 0.05, *η*^2^ = 0.83) and the discrimination between the two responses grew larger as training progressed (*F*_(1,7)_ = 56.79, *p* < 0.05, *η*^2^ = 0.89). Then, rats received the modified blocking design where the two compounds now included a pre-trained stimulus. That is, the novel stimulus S4 was still presented with the pre-trained S1 but S5 was experienced together with the pre-trained S2. As before, the two compounds terminated in the delivery in O1. By employing such design, we were hoping that both compounds would trigger similar amount of responding, as both included a pre-trained stimulus. However, only one them (S1S4) specifically predicted the specific outcome (O1) that was delivered. Thus, we reasoned that blocking would be stronger to S4 than to S5 given that S1 but not S2 had been trained to predict O1. The results are presented in Figure 5F and similar to what was found before, the compounds elicited more responding than the two stimuli (*F*_(1,7)_ = 27.85, *p* < 0.05, *η*^2^ = 0.8), responding to S1S4 and S4 alone did not differ from that of S2S5 and S5 alone (*F* < 2.8) and responding to S4 and S5 depended on whether they were presented in compound or not (*F*_(1,7)_ = 7.312, *p* < 0.05, *η*^2^ = 0.51). Critically, the two compounds elicited the same levels of magazine entries (*F* < 0.14). Yet, S5 was able to trigger more responding than S4 when the stimuli were presented alone (*F*_(1,7)_ = 9.776, *p* < 0.05, *η*^2^ = 0.58). These results indicate that S1 specifically predicted O1 and was therefore able to block the association between that outcome and the co-present S4.

In order to reproduce the results just obtained, rats in the two control groups received additional discrimination training following the test administered after Pavlovian extinction (Figure 5D). One rat (one male) in those two groups was excluded from the analysis as magazine recordings failed during the blocking stage. Additional discrimination training was successful as performance increased across days (*F*_(1,12)_ = 16.68, *p* < 0.05, *η*^2^ = 0.76), responding was higher on the correct response than the incorrect one (*F*_(1,12)_ = 118.86, *p* < 0.05, *η*^2^ = 0.91) and the discrimination between the two responses grew larger as training progressed (*F*_(1,12)_ = 21.62, *p* < 0.05, *η*^2^ = 0.64). The rats were then submitted to the modified blocking design described before (Figure 5A). The results presented in Figure 5G replicated those of rats from group Reverse/Immediate. The compounds elicited more responding than the two stimuli (*F*_(1,12)_ = 14.15, *p* < 0.05, *η*^2^ = 0.54). Performance on S1S4 and S4 alone was lower than that of S2S5 and S5 alone (*F*_(1,12)_ = 8.06, *p* < 0.05, *η*^2^ = 0.4) and responding to S4 and S5 depended on whether they were presented in compound or not (*F*_(1,12)_ = 5.68, *p* < 0.05, *η*^2^ = 0.32). Critically, the two compounds elicited the same levels of magazine entries (*F* < 1.6). Yet, S5 was able to trigger more responding than S4 when the stimuli were presented alone (*F*_(1,12)_ = 8.9, *p* < 0.05, *η*^2^ = 0.32).

## Discussion

The present experiments revealed that rats readily learned the contingencies arranged by initial discrimination training. Across this training, rats used the stimuli to select the response that was reinforced (i.e., the correct response) as opposed to the one that was not reinforced (i.e., the incorrect response). Learning was further confirmed during a subsequent choice test conducted under extinction where performance on the correct response was greater than that on the incorrect response. This choice test also showed that the initial contingencies were encoded in an outcome-specific manner. Devaluing the outcome that the correct response used to earn significantly reduced performance on that response. Thus, rats not only learned that the stimuli signaled which response was delivering food, they also learned information about the sensory-specific properties of the delivered food. The rats were also able to detect and adjust to the reversal of the initial contingencies. Once again, this was evident during reversal training and the subsequent choice test during which correct responding was higher than incorrect responding. Furthermore, the devaluation manipulation revealed that the reversed contingencies, just like the initial ones, were encoded in an outcome-specific manner. It should be noted, however, that the initial contingencies interfered with the expression of the reversed contingencies (Figures 3H–J). Yet, this interference remained negligible compared to that observed when a delay was inserted between reversal training and the choice test. During that delayed test, rats displayed slightly higher performance on the correct response than the incorrect one but they were unable to use the sensory-specific associations of the outcomes to appropriately direct their behavior. Importantly, the second experiment showed that this impairment was specific to the delay inserted between reversal training and test. The present findings are therefore consistent with the view that reversal learning imposes inhibition upon the initial contingencies and that this inhibition wanes with the passage of time.

Our experiments demonstrated that the passage of time significantly interfered with the behavior produced by reversal training. When tested immediately after this training, rats successfully used the reversed contingencies to choose the instrumental response in an outcome-specific manner. This outcome-specificity was lost when a delay was inserted between reversal training and test, such that rats distributed equally their performance on the instrumental responses regardless of the value attributed to the outcome that they used to earn. Yet, the chosen responses remained those that were reinforced across reversal training. Although this finding confirms an important role for the passage of time, it is at odds with previous studies showing that rats switch back to using the initial contingencies when behavior is assessed several days after reversal training (Rescorla, 2007; Scarlet et al., 2009). One obvious explanation for such discrepancy is that the delay employed in the current experiments was not sufficient to restore the control exerted by the initial contingencies over behavior. However, this explanation appears unlikely, as the time interval used in our experiments sits right in between the ones imposed in previous studies. Thus, it seems more likely that methodological differences are responsible for the discrepant findings. In his study, Rescorla (2007) employed a design where the two stimuli were presented alone either in the first or the second stage. As mentioned previously, such design involves explicit extinction training and potentially raises a role for latent inhibition. Further, it does not provide any means to test whether the content of the learning driving behavior is outcome-specific or not. This outcome-specificity was however examined in the study conducted by Scarlet et al. (2009). Yet, this particular study used flavor conditioning to investigate reversal learning and there are known differences between that form of conditioning and the appetitive conditioning procedure used in the current study. Critically, these differences are especially evident when assessing sensory-specific associations and the impact of extinction on such associations (Rescorla, 1996; Delamater, 2007a). Regardless, we found that the passage of time significantly disrupted the behavior generated by reversal training, such that animals were unable to use the outcome-specific content of that training to choose between instrumental responses.

The crucial role played by the passage of time was only evident when assessing whether the behavior was controlled by outcome-specific information. Indeed, the delayed tests in both experiments revealed higher responding on the reversed contingencies, suggesting that these contingencies remained dominant during these tests. The requirement for testing outcome-specific content to highlight the role of time in reversal learning is somewhat surprising if the original assumption is that this role reflects the involvement of extinction-like processes. For instance, Pavlovian-related tasks have shown that extinction leaves sensory-specific associations relatively unaffected. This is particularly clear in specific Pavlovian-instrumental transfer (PIT) tasks that include a Pavlovian stage during which two stimuli are trained to predict two specific outcomes and an instrumental stage during which the two outcomes can be earned by performing one of two distinct responses. A subsequent choice test typically reveals that a stimulus predicting a particular outcome biases choice towards the response earning that same outcome (Colwill and Rescorla, 1988). Critically, this bias is left intact by extinction of the stimulus-outcome associations, even though the stimuli lose their ability to elicit Pavlovian responding (Delamater, 1996; Laurent et al., 2016). Provided such findings, we would have expected time to firstly interfere with choice based on the reinforcement history of the responses (i.e., Correct vs. Incorrect) rather than choice based on the sensory-specific associations that these responses had with their outcomes. This was clearly not the case in the present experiments. Thus, we propose that reversal learning differs from extinction learning in its ability to alter outcome-specific associations but that the expression of both forms of learning is highly sensitive to the passage of time. Over and above this proposal, our experiments clearly indicate that a better understanding of the mechanisms underlying reversal training requires an adequate assessment of the content of the learning that it produces.

So far, we have discussed differences between our results and those from previous studies. One reason for such singularity is to assume that the associative constructs underlying performance in our task were distinct from those implicated in previous studies. This assumption is certainly valid, as evidence indicates that various associations may have contributed to the behavior observed in the current experiments (Colwill and Rescorla, 1990; Bradfield and Balleine, 2013). In that respect, we can reasonably conclude that simple response-outcome associations were not the main driver of the behavior observed, as responding in the absence of the stimuli was negligible throughout the various stages of our experiments. It is, however, tempting to argue that our task was driven by associations similar to those producing the specific PIT effect. The motivation for such argument is that just like specific PIT, responding in our experiments remained insensitive to extinction of the Pavlovian associations. However, this responding was strongly influenced by the value attributed to the outcomes and specific PIT is known to be resistant to outcome devaluation and even to changes in primary motivational states (Holland, 2004; Corbit et al., 2007). Thus, it appears that the stimulus-response or stimulus-outcome-response associations that have been proposed to mediate specific PIT (Balleine and Ostlund, 2007; Cohen-Hatton et al., 2013) were not the ones sustaining performance in the present experiments. This performance could however have been supported by so-called hierarchical associations (Colwill and Rescorla, 1990; Bradfield and Balleine, 2013). Among these, our experiments appear to favor a role for (stimulus-outcome)-response associations where the performance of the response is controlled by the specific stimulus-outcome associations and the outcome value (Rescorla, 1992b). One reason for favoring this associative structure is the strong influence of outcome devaluation in our tasks. Another reason is that, in both experiments, we found that the trained stimuli were able to block appetitive conditioning to another stimulus. It must be acknowledged that a previous study did fail to obtain blocking using a stimulus trained to signal that a response delivers a food outcome (Holman and Mackintosh, 1981). However, this study did not employ a discrimination procedure, it did not administer reversal training and blocking was assessed against control stimuli that were distinct from those used in our experiments. It is noteworthy however, that the authors did acknowledge that their results were not excluding the possibility that discriminative stimuli are able to produce blocking. Rather, their findings merely indicate that the blocking they produce is not as large as the one generated by pure Pavlovian stimuli. Regardless, our experiments revealed that the trained stimuli were able to produce appetitive blocking in an outcome-specific manner, suggesting that specific stimulus-outcome associations were critical in generating the performance following initial discrimination training and reversal training.

In summary, the present experiments demonstrate that the passage of time interferes with adaptive behavior following reversal learning. When tested immediately after reversal training, rats were able to use the stimuli to select the response earning a valued outcome as opposed to the response delivering a devalued outcome. This outcome-specific choice produced by the stimuli was abolished when it was assessed during a test conducted several days after reversal training. This interference distinguishes reversal learning from extinction learning, as the latter has typically been found to have little impact on outcome-specific information. However, both reversal and extinction learning are sensitive to the passage of time. Further studies are required to determine if the present findings extend to tasks where other associative constructs (e.g., in pure Pavlovian tasks) govern behavior. Regardless, our findings highlight the need for assessing outcome-specific information to understand how reversal learning influences adaptive behavior.

## Author Contributions

EHFG and VL designed the experiments and analyzed the data. EHFG, NWL and VL wrote the manuscript. EHFG conducted the experiments.

## Conflict of Interest Statement

The authors declare that the research was conducted in the absence of any commercial or financial relationships that could be construed as a potential conflict of interest.
